# Inhibition of the prostaglandin D_2_–GPR44/DP2 axis improves human islet survival and function

**DOI:** 10.1007/s00125-020-05138-z

**Published:** 2020-04-29

**Authors:** Shadab Abadpour, Björn Tyrberg, Simen W. Schive, Charlotte Wennberg Huldt, Peter Gennemark, Erik Ryberg, Tina Rydén-Bergsten, David M. Smith, Olle Korsgren, Stanko Skrtic, Hanne Scholz, Maria Sörhede Winzell

**Affiliations:** 1 Department of Transplant Medicine and Institute for Surgical Research, Oslo University Hospital, Sognsvannsveien 20, 0027 Oslo, Norway; 2 Hybrid Technology Hub, Centre of Excellence, Institute of Basic Medical Sciences, University of Oslo, Oslo, Norway; 3 Research and Early Development, Cardiovascular, Renal and Metabolism, BioPharmaceuticals R&D, AstraZeneca, Peppredsleden 1, 431 83 Mölndal, Gothenburg, Sweden; 4 Department of Biomedical Engineering, University of Linköping, Linköping, Sweden; 5 Hit Discovery, Discovery Sciences, BioPharmaceuticals R&D, AstraZeneca, Cambridge, UK; 6 Department of Immunology, Genetics and Pathology, Science for Life Laboratory, University of Uppsala, Uppsala, Sweden; 7 Institute of Medicine at Sahlgrenska Academy, University of Gothenburg, Gothenburg, Sweden

**Keywords:** DP2, GPR44, Human islets, Islet apoptosis, Islet survival rate, Prostaglandin D_2_

## Abstract

**Aims/hypothesis:**

Inflammatory signals and increased prostaglandin synthesis play a role during the development of diabetes. The prostaglandin D_2_ (PGD_2_) receptor, GPR44/DP2, is highly expressed in human islets and activation of the pathway results in impaired insulin secretion. The role of GPR44 activation on islet function and survival rate during chronic hyperglycaemic conditions is not known. In this study, we investigate GPR44 inhibition by using a selective GPR44 antagonist (AZ8154) in human islets both in vitro and in vivo in diabetic mice transplanted with human islets.

**Methods:**

Human islets were exposed to PGD_2_ or proinflammatory cytokines in vitro to investigate the effect of GPR44 inhibition on islet survival rate. In addition, the molecular mechanisms of GPR44 inhibition were investigated in human islets exposed to high concentrations of glucose (HG) and to IL-1β. For the in vivo part of the study, human islets were transplanted under the kidney capsule of immunodeficient diabetic mice and treated with 6, 60 or 100 mg/kg per day of a GPR44 antagonist starting from the transplantation day until day 4 (short-term study) or day 17 (long-term study) post transplantation. IVGTT was performed on mice at day 10 and day 15 post transplantation. After termination of the study, metabolic variables, circulating human proinflammatory cytokines, and hepatocyte growth factor (HGF) were analysed in the grafted human islets.

**Results:**

PGD_2_ or proinflammatory cytokines induced apoptosis in human islets whereas GPR44 inhibition reversed this effect. GPR44 inhibition antagonised the reduction in glucose-stimulated insulin secretion induced by HG and IL-1β in human islets. This was accompanied by activation of the Akt–glycogen synthase kinase 3β signalling pathway together with phosphorylation and inactivation of forkhead box O-1and upregulation of pancreatic and duodenal homeobox-1 and HGF. Administration of the GPR44 antagonist for up to 17 days to diabetic mice transplanted with a marginal number of human islets resulted in reduced fasting blood glucose and lower glucose excursions during IVGTT. Improved glucose regulation was supported by increased human C-peptide levels compared with the vehicle group at day 4 and throughout the treatment period. GPR44 inhibition reduced plasma levels of TNF-α and growth-regulated oncogene-α/chemokine (C-X-C motif) ligand 1 and increased the levels of HGF in human islets.

**Conclusions/interpretation:**

Inhibition of GPR44 in human islets has the potential to improve islet function and survival rate under inflammatory and hyperglycaemic stress. This may have implications for better survival rate of islets following transplantation.

**Electronic supplementary material:**

The online version of this article (10.1007/s00125-020-05138-z) contains peer-reviewed but unedited supplementary material, which is available to authorised users.

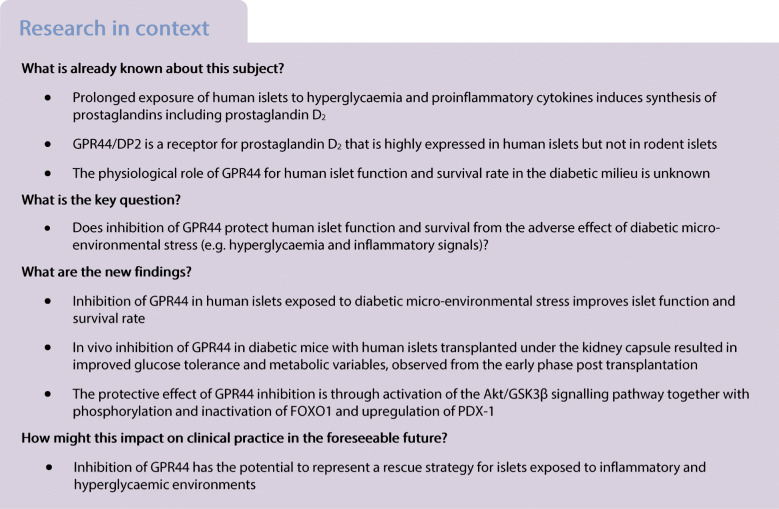



## Introduction

GPR44 (also known as PTGDR2, DP2 or CRTh2) is a transmembrane G-protein coupled receptor for prostaglandin D_2_ (PGD_2_), shown to be highly expressed in human beta cells through proteomics screening analysis [1]. Furthermore, it has been identified as a useful imaging biomarker for visualising of human beta cell mass [2]. The physiological role of GPR44 in human islet function and survival in the diabetic milieu is however unknown.

One hallmark of both type 1 and type 2 diabetes is the loss of functional beta cell mass resulting in an insufficient release of insulin and development of hyperglycaemia [3]. Prolonged hyperglycaemia has an adverse effect on pancreatic beta cell function and mass due, at least partly, to the activation of proinflammatory responses leading to beta cells apoptosis [4]. Proinflammatory prostaglandins are lipid molecules derived from arachidonic acid in cell-membrane phospholipids and their synthesis is triggered by inflammatory signals [5]. IL-1β, a proinflammatory cytokine known to induce beta cell injury and loss, has been found to increase prostaglandin production in islets through activation of NF-κB [6].

PGD_2_ signals through two G-protein coupled receptors, GPR44 and DP1 [7], the former being highly expressed in human islets [1]. Although, there is not much known about the role of PGD_2_ and the regulatory mechanism of PGD_2_–GPR44 in human islets, it has been demonstrated that PGD_2_ is synthesised and secreted most likely by the pancreatic stellate cells in human islets [8] and that elevated levels of glucose and IL-1β increase PGD_2_ synthesis in vitro in rat and human islets in a similar fashion as previously described for prostaglandin E_2_ (PGE_2_) [8, 9]. Furthermore, PGD_2_ signalling in immune cells via GPR44 couples to inhibitory G-proteins resulting in reduction of intracellular cAMP [10]. This suggests that the activation of GPR44 in beta cells could also result in reduction of cAMP and inhibition of glucose-stimulated insulin secretion. The same observation has been previously reported with PGE_2_ [5, 8]. Significant reduction of GPR44 protein expression has been noted in insulin-negative human islets in pancreatic sections from individuals with long-standing type 1 diabetes [1]. In contrast, the expression of this receptor was upregulated in islets from individuals with type 2 diabetes compared with healthy individuals [11]. Taken together, these observations suggest that the activation of GPR44 may have a negative impact on islet function in an inflammatory and hyperglycaemic state and that inhibition of GPR44 may improve islet function and survival rate.

In the present study, we used the specific GPR44 antagonist AZ8154 to investigate the role of GPR44 in the function and survival rate of human islets exposed to a type 1 diabetes-like milieu both in vitro (mimicked by islet exposure to high concentration of glucose [HG] and to IL-1β) and in vivo (using human islets transplanted to a diabetic immunodeficient mouse model). We also explored the intracellular responses associated with GPR44 inhibition in isolated human islets in vitro.

## Methods

### Animal studies

All experiments and methods using human islets were approved by and performed in accordance with the guidelines and regulations made by the regional committee for medical and health research ethics central in Norway (2011/782). The animal experiments were approved by the Norwegian National Animal Research Authority (FOTS ID 7005) and were performed in accordance with the European Directive 2010/63/EU and The Guide for the Care and Use of Laboratory Animals, 8th edition (NRC 2011, National Academic Press) or by the Local Ethics Review Committee for Animal Experiments (Gothenburg, Sweden). The AstraZeneca Gothenburg animal unit is accredited by the Association for the Assessment and Accreditation of Laboratory Animal Care (AAALAC).

### Compounds and in vitro pharmacology

The GPR44 antagonist AZ8154 is an analogue to the previously published GPR44 antagonist AZD1981, 4-(acetylamino)-3-[(4-chlorophenyl)thio]-2-methyl-1*H*-indole-1-acetic acid [12], a clinical drug candidate intended for the treatment of asthma [13, 14]. Both compounds were synthesised by AstraZeneca and came from two neighbouring chemical series. Potencies of AZ8154 and AZD1981 on binding to human and mouse GPR44 and DP1 receptors (electronic supplementary material [ESM] Methods, ESM Table 1) and inhibition of GPR44 receptor on EndoC-βH1 cells (human beta cell line, Univercell Biosolutions, Toulouse, France [mycoplasma negative]) using insulin secretion and cAMP measurement assays (ESM Table 2, ESM Fig. 1) are described in detail in the ESM Methods. Selectivity analysis of AZD1981 and AZ8154 on the PGE_2_ receptor, EP3 shows that neither of the AZ8154 and AZD1981 antagonists bound to EP3 in human primary adipocytes and therefore did not have an inhibitory effect on EP3 (ESM Methods and ESM Results/EP3 selectivity for AZD1981 and AZ8154, ESM Fig. 2). AZ8154 was chosen over AZD1981 for this study due to its improved pharmacokinetic profile in mice (ESM Methods, ESM Figs. 3, 4).

### Human islet culture and experimental condition

Human islets from non-diabetic donors were obtained from Prodo laboratory (Aliso Viejo, CA, USA). Islets were dispersed and exposed to a stable PGD_2_ analogue, 15(*R*)-15-methyl-PGD_2_ (Cayman Chemicals, Ann Arbor, MI, USA), at the concentrations 1, 10 or 100 nmol/l, or to a mixture of the cytokines IL-1β (10 ng/ml), IFN-γ (50 ng/ml) and TNF-α (50 ng/ml) (R&D systems, Minneapolis, MN, USA) with or without addition of AZ8154 (1, 3 or 10 μmol/l), to evaluate the effect of the ligand PGD_2_ and the GPR44 antagonist on islet apoptosis.

Human islets were obtained from the JDRF award 31-2008-416 (ECIT Islet for Basic Research program) and isolated as previously described [15] from male/female 7/3 non-diabetic brain-dead donors with mean age 55 years (35–69 years) and mean BMI 24 kg/m^2^ (23–42 kg/m^2^) after appropriate informed consent from relatives for multi-organ donation and for use in research. A human islet checklist is provided in the ESM. All experiments and methods using human islets were approved by and performed in accordance with the guidelines and regulations made by regional committee for medical and health research ethics central in Norway (2011/782). An islet purity of >50% was used in this study, judged by digital imaging analysis [16] or dithizone staining. Equally sized islets were manually hand-picked and distributed blindly among experimental groups. Islets were cultured at 37°C (5% CO_2_) for 48 h in petri dishes (Sterilin, Newport, UK) with CMRL 1066 medium supplemented with 2% human AB serum (Milan ANALYTICA, Rheinfelden, Switzerland), 1% penicillin/streptomycin, 10 mmol/l HEPES (Life Technologies, Carlsbad, CA, USA) without (untreated islets) or with a combination of HG (20 mmol/l glucose) and IL-1β (10 ng/ml) (R&D systems, Minnneapolis, MN, USA) with or without AZ8154 (10 μmol/l).

### Cell death and apoptosis analysis

Programmed cell death and the level of caspase activity was analysed in human islets by using the Cell Death Detection ELISA^PLUS^ kit (Roche Diagnostics, Mannheim, Germany) and Caspase-Glo 3/7 assay (Promega Biotech, Madison, WI, USA). See ESM Methods for details.

### Glucose-stimulated insulin secretion assay

Human islets were incubated in 1.67 mmol/l glucose for 45 min at 37°C followed by 45 min incubation with 20 mmol/l glucose. Insulin secretion was analysed in the supernatant fraction using human insulin enzyme immunoassay (EIA) (Mercodia, Uppsala, Sweden). See ESM Methods for details.

### Islet transplantation and rationale for dose selection of the GPR44 antagonist

Diabetes was introduced in 8- to 10-week-old male NMRI-nude HsdCpb:NMRI-*Foxn1*^nu^ mice (Harlan, Indianapolis, IN, USA) by one intravenously administered dose of Alloxan monohydrate 75 mg/kg (Sigma Aldrich, Oslo, Norway) 3 days prior to islet transplantation. Mice with non-fasted blood glucose ≥20 mmol/l for two consecutive days were selected as diabetic recipients. In all experiments, 500 hand-picked human islets were transplanted under the left kidney capsules as previously described [17]. Transplantation was performed blinded as the surgeon did not know the design of the experiments. Mice were randomly selected into groups and treated with the GPR44 antagonist AZ8154 or vehicle (0.5% hydroxypropyl methyl cellulose (wt/vol.) [HPMC]) 1 day prior to islet transplantation. Mouse body weights were measured every other day until termination of the studies. Mice were divided into three cohorts. The first cohort consisted of eight diabetic mice transplanted with human islets from one donor. These mice were administered orally with 100 mg/kg per day of AZ8154 up to day 17 post transplantation. As control, eight mice received vehicle treatment. The dose of AZ8154 was chosen to achieve full inhibition of GPR44 over 24 h (more details can be found in the ESM Methods/Pharmacokinetic analysis of AZ8154 and AZD1981, ESM Results/Pharmacokinetic evaluation of AZ8154 for determination of dose in the human islet transplantation studies and ESM Fig. 4a,b). The second cohort consisted of 24 mice transplanted with human islets. These mice were divided into three groups and a dose–response study was performed with two doses of AZ8154 (6 mg/kg per day [*n* = 8] and 60 mg/kg per day [*n* = 8], or vehicle [HPMC, *n* = 8]) for 17 days. The low dose of AZ8154 was set to achieve exposure in the range of the in vitro IC_50_ value, while the high dose was chosen to achieve maximal inhibition over 24 h (more details can be found in the ESM Methods/Pharmacokinetic analysis of AZ8154 and AZD1981, ESM Results/Pharmacokinetic evaluation of AZ8154 for determination of dose in the human islet transplantation studies and ESM Fig. 4a,c). The third cohort of mice was used to investigate the effect of GPR44 inhibition in vivo on the early phase post transplantation. Twenty-eight mice transplanted with human islets from two donors were treated with 60 mg/kg per day of AZ8154 (*n* = 14) or vehicle (*n* = 14). The experiment was terminated 4 days post transplantation to explore signalling pathways that were activated following GPR44 inhibition. Islet grafts were collected and immediately snap-frozen in liquid nitrogen and stored at −70°C until further gene and protein expression analysis. No animals were excluded from the studies.

### Glucose measurements and IVGTT

Human islet response to glucose was investigated by performing an IVGTT on day 10, 4 h after administration of the GPR44 antagonist to the mice, and on day 15 without the morning administration of the GPR44 antagonist. See ESM Methods for details.

### Biochemical measurements

In vivo human-specific C-peptide, proinsulin, insulin, TNF-α and growth-regulated oncogene-α (GRO-α) were measured in plasma samples. The protein level of hepatocyte growth factor (HGF), phosphorylated Akt and glycogen synthase kinase 3β (GSK3β) were measured in the human islet lysate. See ESM Methods for details.

### Immunofluorescent staining

At termination of the in vivo studies, the entire grafts were harvested and fixed in 10% formalin, embedded in paraffin and sectioned for immunofluorescent staining of insulin. Slides were scanned and images were taken by the slide scanner Axioscan Z1 (Carl Ziess, Oberkochen, Germany) operated by the ZEN lite blue software. See ESM Methods for details.

### Real-time qPCR

Frozen islet grafts were homogenised, followed by total RNA isolation. TaqMan primers and probes were used for mRNA analysis of human *MAFA*, *PDX-1*, *TNF-α* (also known as *TNF*) and *GRO-α* (also known as *CXCL1*). Results were normalised to the housekeeping gene human *RPL30*. See ESM Methods for details.

### Western blot analysis

Equal amounts of total protein (20 μg) were analysed for phosphorylated forkhead box O-1 (FOXO1), total FOXO1, pancreatic and duodenal homeobox-1 (PDX-1) and GAPDH. See ESM Methods for details.

### Statistical analysis

Data are presented as means ± SD. Differences among the three groups were evaluated by one-way ANOVA with Bonferroni corrections. A Mann–Whitney *U* test was performed for difference analysis between two groups. Significance was set at *p* < 0.05. Data were analysed using GraphPad Prism software, version 6.0 (La Jolla, CA, USA).

## Results

### GPR44 inhibition using the antagonist AZ8154 reverses PGD_2_-induced apoptosis and protects human islet function under type 1 diabetes-like milieu

**Fig. 1:**
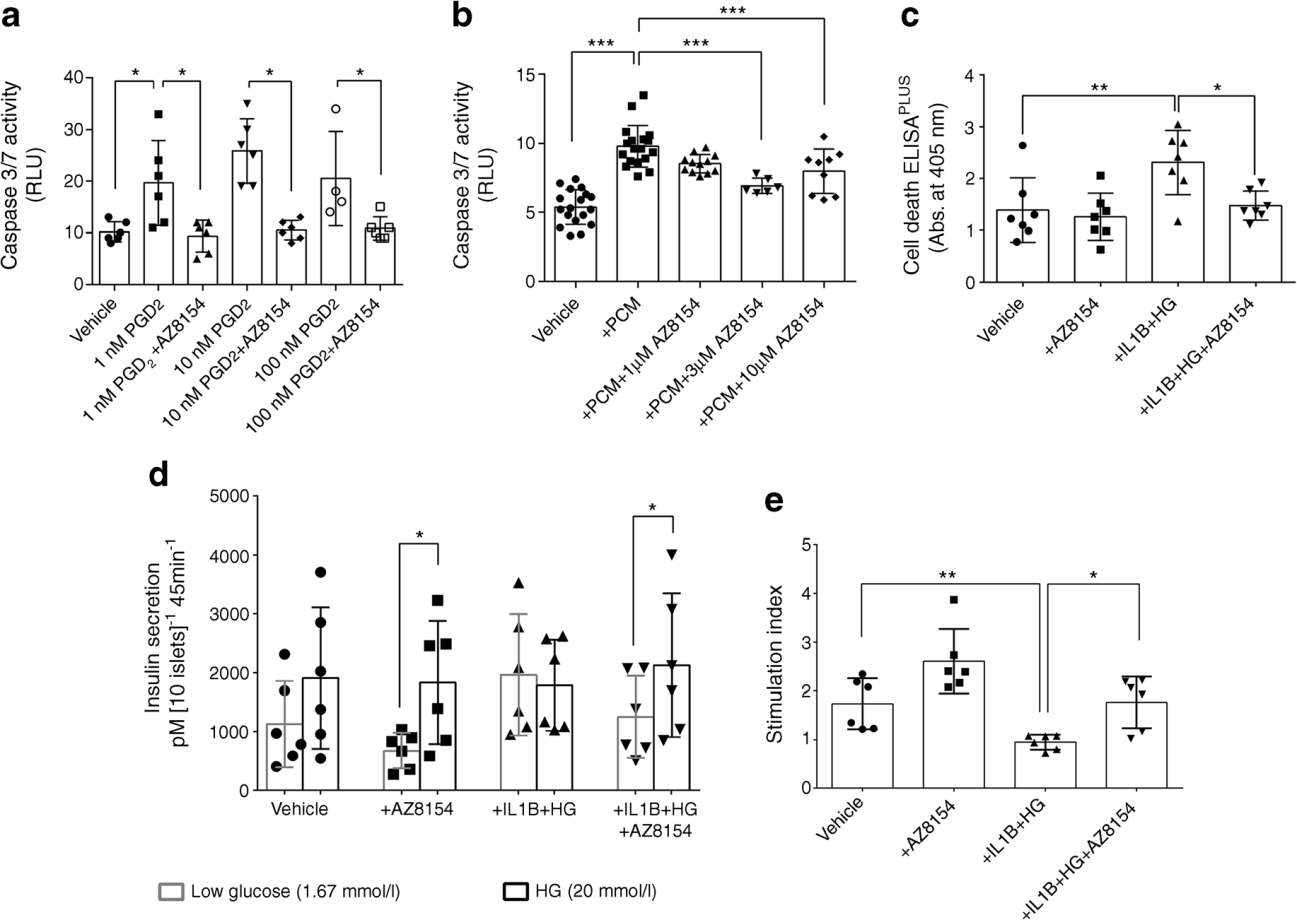
Inhibition of GPR44 results in reduced apoptosis and improved function in human islets. (**a**) Caspase 3/7 activity in human islets treated with 15(*R*)-15-methyl-PGD_2_ (1, 10 and 100 nmol/l) with or without AZ8154 (10 μmol/l) for 24 h, *n* = 3, with each incubation condition run in duplicate. (**b**) Caspase 3/7 activity in human islets treated with a proinflammatory cytokine mix (10 ng/ml IL-1β, 50 ng/ml TNF-α and 50 ng/ml INF-γ) with or without AZ8154 (1, 3 and 10 μmol/l) for 24 h, *n* = 3–9, with each incubation condition run in duplicate. (**c**) Apoptosis evaluated in isolated human islets treated with either AZ8154 (10 μmol/l) or HG (20 mmol/l) + IL-1β (10 ng/ml) with or without AZ8154 (10 μmol/l) for 48 h, *n* = 7 individual donors. (**d**, **e**) Insulin secretion in response to basal (1.67 mmol/l) and stimulated (20 mmol/l) levels of glucose measured by EIA (**d**) and calculated as stimulation index (**e**) for human islets treated with either AZ8154 (10 μmol/l) or HG (20 mmol/l) + IL-1β (10 ng/ml) with or without AZ8154 (10 μmol/l) for 48 h, *n* = 6 independent donors. In all analyses, data are presented as means ± SD and analysed by one-way ANOVA with Bonferroni corrections. *p < 0.05, **p < 0.01 and ***p < 0.001; Abs., absorbance; PCM, proinflammatory cytokine mix (IL1-β, TNF-α and INF-γ); RLU, relative light units

Both GPR44 antagonists, AZ8154 and AZD1981, were equally potent in blocking PGD_2_ receptors on human and mouse HEK293 cells overexpressing PGD_2_ receptors, GPR44 and DP1 (ESM Results, ESM Table 1). Both antagonists were also potent in inhibition of cellular responses mediated by GPR44 receptor in human EndoC-βH1 cells (ESM Results, ESM Table 2, ESM Fig. 1a,d). The stable PGD_2_ analogue 15(*R*)-15-methyl-PGD_2_ potently induced apoptosis in dispersed human islets, with maximal caspase 3/7 activity detected at 1 nmol/l PGD_2_ (Fig. 1a). Addition of the specific GPR44 antagonist AZ8154 (10 μmol/l) significantly reduced the caspase 3/7 activity back to basal levels at all three tested concentrations (1, 10, 100 nmol/l) of 15(*R*)-15-methyl-PGD_2_ (Fig. 1a). Furthermore, treatment with the GPR44 antagonist significantly reduced caspase 3/7 activity induced by proinflammatory cytokine mix (IL-1β, TNF-α and INF-γ) but did not normalised the activity to basal levels (Fig. 1b). We demonstrated increased cell death in human islets treated with HG + IL-1β for 48 h (Fig. 1c), and the GPR44 antagonist significantly reduced the level of cell death induced by HG + IL-1β (Fig. 1c).

HG + IL-1β reduced insulin secretion in response to HG in human islets by increasing the basal insulin secretion and consequently reduced the stimulation index compared with the untreated islets (Fig. 1d,e). The adverse effect of HG + IL-1β on insulin secretion was reversed by GPR44 antagonist treatment (Fig. 1d,e).

### GPR44 inhibition in vivo improves glucose tolerance in diabetic mice transplanted with a marginal dose of human islets

**Fig. 2:**
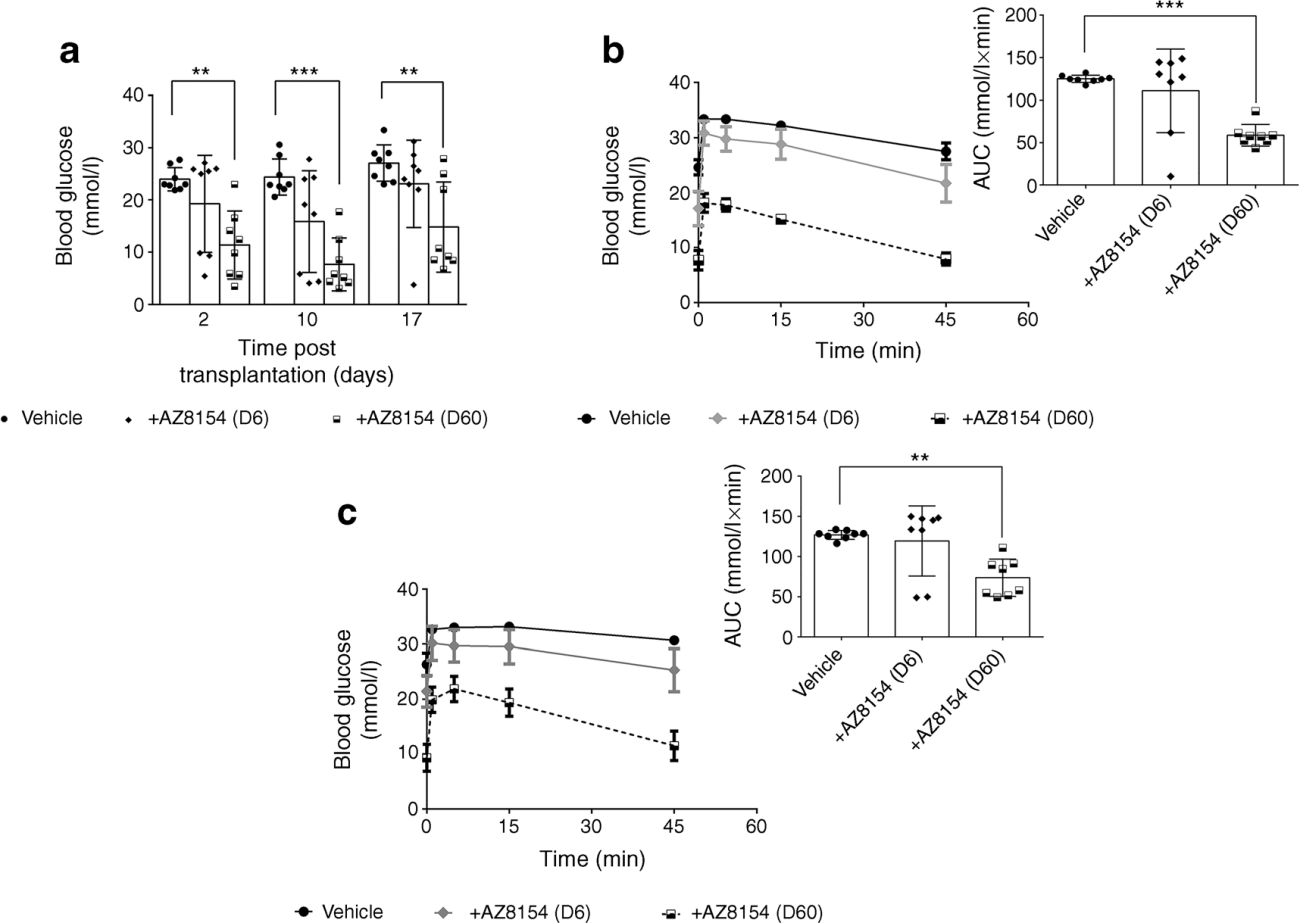
GPR44 inhibition enhances glucose tolerance of human islets transplanted in diabetic mice. (**a**) Fasting blood glucose was measured on day 2, 10 and 17 in mice transplanted with human islets and treated with 6 or 60 mg/kg per day of AZ8154 or vehicle. (**b**, **c**) Blood glucose levels during IVGTT and the corresponding AUCs represent the difference in the level of glucose in mice transplanted with human islets and treated with AZ8154 or vehicle on day 10, 4 h after administration of AZ8154 (**b**) and on day 15, 18 h after administration of AZ8154 (**c**). Data are presented as means ± SD. The differences in AUC corresponding to the glucose levels during IVGTT were analysed with one-way ANOVA with Bonferroni corrections. **p < 0.01 and ***p < 0.001. For all analysis *n* = 8 mice/experimental group. D6, 6 mg/kg per day; D60, 60 mg/kg per day

Diabetic mice bearing human islet grafts showed significant improvement in fasting blood glucose measured on day 2 and day 17 post transplantation following treatment with GPR44 antagonist (60 mg/kg per day) vs vehicle (Fig. 2a). In response to an intravenous glucose challenge performed on day 10, the mice treated with 60 mg/kg per day of the GPR44 antagonist AZ8154 showed significantly improved glucose tolerance compared with the vehicle group as shown by AUC quantitative analysis of the glucose excursion curves (Fig. 2b). To exclude acute effects of the GPR44 antagonist on improved glucose response in islets, an IVGTT was performed on day 15 post transplantation after an 18 h washout period of AZ8154. The blood glucose AUC was significantly improved in mice treated with 60 mg/kg per day of the GPR44 antagonist AZ8154 compared with vehicle (Fig. 2c). The low dose of AZ8154 (6 mg/kg per day) affected neither the fasting blood glucose nor the glucose tolerance (Fig. 2a–c). Body weight measurement every other day throughout the study showed no significant difference among the groups (data not shown).

### In vivo GPR44 inhibition preserves transplanted human islet grafts

**Fig. 3:**
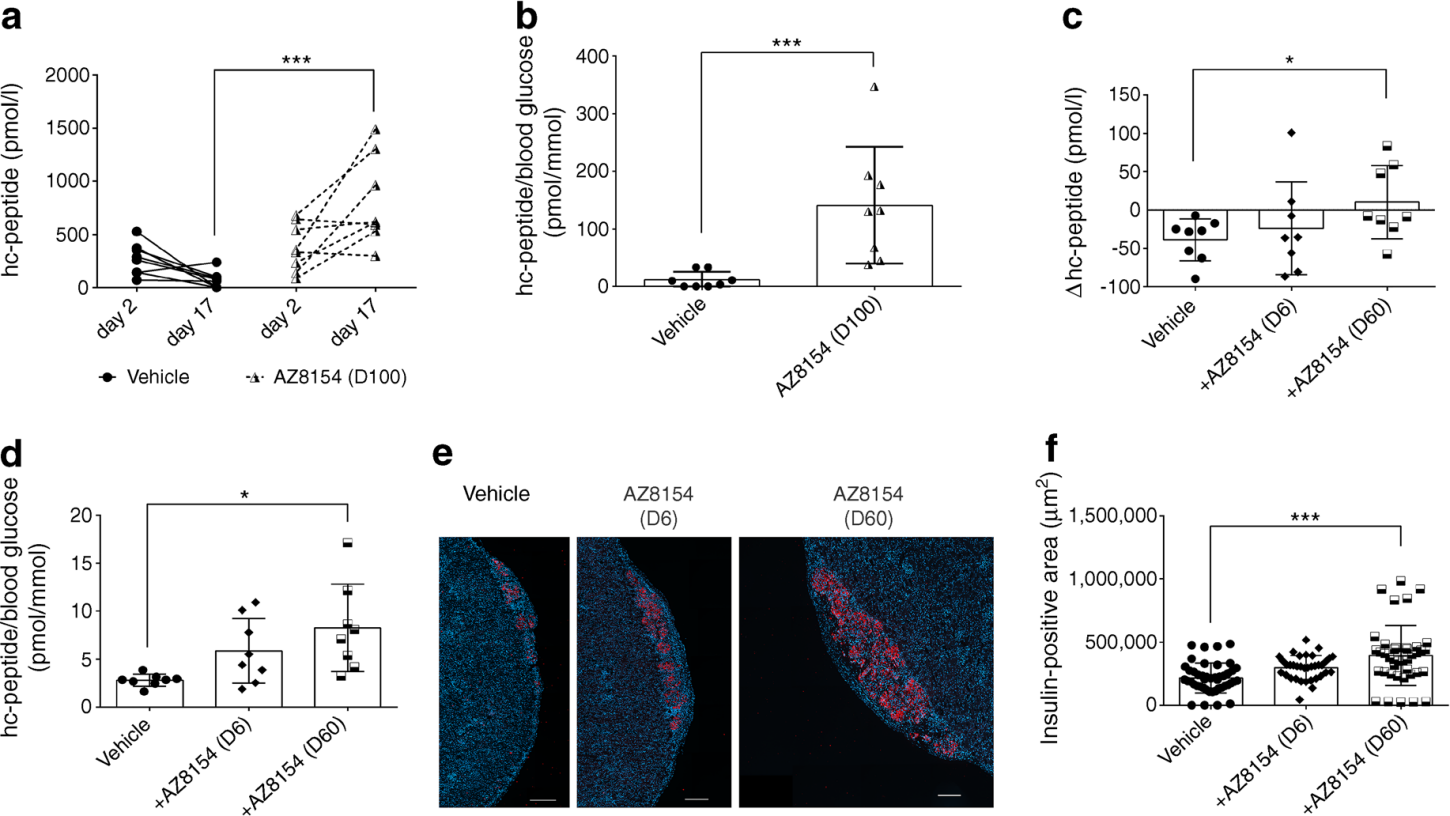
GPR44 inhibition improves human islet function and preserves beta cell mass post transplantation in diabetic mice. (**a**) Human C-peptide levels measured on day 2 and day 17 in mice transplanted with human islets and treated with 100 mg/kg per day AZ8154 or vehicle, *n* = 8 mice/each experimental group. (**b**) The ratio of human C-peptide over fasting blood glucose analysed on day 17 in mice transplanted with human islet grafts and treated with 100 mg/kg per day AZ8154 or vehicle, *n* = 8 mice/each experimental group. (**c**) Assessment of difference (Δ value) in the level of plasma human C-peptide measured on day 2 and day 17 in mice transplanted with human islets and treated with 60 mg/kg per day AZ8154 or vehicle, *n* = 8 mice/each experimental group. (**d**) The ratio of human C-peptide over fasting blood glucose analysed on day 17 in mice transplanted with human islet grafts and treated with 60 mg/kg per day AZ8154 or vehicle, *n* = 8 mice/each experimental group. (**e**, **f**) Representative images showing immunofluorescent staining of insulin (red) together with DAPI nuclear staining (blue) on day 17 (**e**) and quantification of the area of insulin-positive cells within transplanted islet grafts (**f**) treated with AZ8154 or vehicle. Magnification ×10; scale bar, 200 μm. In all analyses, data are presented as means ± SD and analysed with one-way ANOVA with Bonferroni corrections. *p < 0.05 and ***p < 0.001. D6, 6 mg/kg per day; D60, dose 60 mg/kg per day; D100, 100 mg/kg per day; hc-peptide, human C-peptide

Circulating human C-peptide measured on day 2 and 17 post transplantation in diabetic mice was significantly improved in mice treated with 100 mg/kg per day AZ8154 vs vehicle (Fig. 3a). Furthermore, the ratio of human C-peptide over fasting blood glucose was increased in mice treated with 100 mg/kg per day AZ8154 vs vehicle (Fig. 3b).

We also observed a significant increase in human C-peptide levels in mice treated with 60 mg/kg per day of AZ8154 vs vehicle (Fig. 3c), calculated as the difference (Δ value) between day 2 and day 17 post transplantation. In addition, the ratio of human C-peptide over fasting blood glucose was increased in mice treated with 60 mg/kg per day AZ8154 vs vehicle on day 17 post transplantation (Fig. 3d). The transplanted human islet grafts analysed for insulin at termination of the study showed a significant increase in insulin-positive area in mice treated with 60 mg/kg per day AZ8154 vs vehicle (Fig. 3e,f). The lower dose of AZ8154 (6 mg/kg per day) had no effect on preservation of the islet graft function or insulin area. The plasma concentration of AZ8154 was measured at the end of the experiment (ESM Figs. 3, 4a–c). For the low dose (6 mg/kg per day), the concentration of AZ8154 only reached sufficient exposure levels for a few hours, which was not sufficient to achieve any treatment effects. It is suggested that full inhibition of GPR44 is needed to protect the islet function.

### In vivo GPR44 inhibition protects human islets from inflammatory responses

**Fig. 4:**
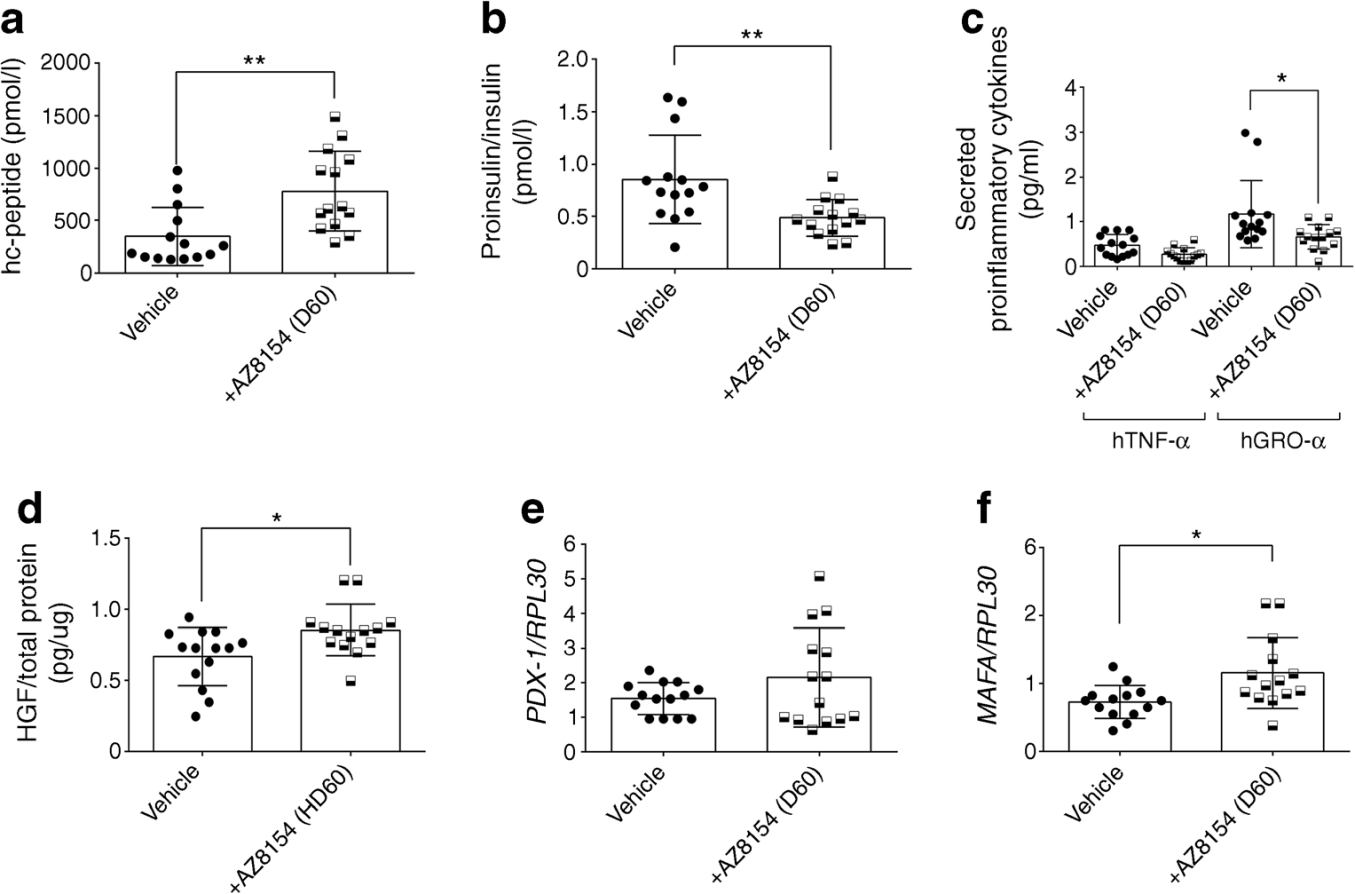
GPR44 inhibition improves islet function and ameliorates secretion of proinflammatory cytokines in early phase post transplantation. (**a**–**c**) Human C-peptide (**a**), ratio of human proinsulin over insulin (**b**) and plasma levels of the human proinflammatory cytokines TNF-α and GRO-α (**c**) analysed on day 4 post transplantation in mice transplanted with human islets and treated with 60 mg/kg per day AZ8154 or vehicle. (**d**–**f**) Human HGF protein level (**d**) and mRNA expression of *PDX-1* (**e**) and *MAFA* (**f**), normalised to *RPL30*, in transplanted human islet lysates obtained from mice treated with AZ8154 60 mg/kg per day or vehicle on day 4 post transplantation. For all analyses *n* = 14 mice/experimental group; data are presented as mean ± SD and analysed by Mann–Whitney *U* test. *p < 0.05 and **p < 0.01. D60, 60 mg/kg per day; hGRO-α, human GRO-α; hc-peptide, human C-peptide; hTNF-α, human TNF-α

Levels of human C-peptide measured 4 days post transplantation were increased in mice treated with 60 mg/kg per day of AZ8154 vs vehicle (Fig. 4a). In addition, the ratio of proinsulin to insulin was lower in mice treated with AZ8154 vs vehicle (Fig. 4b). The mRNA expression of *TNF-α* but not *GRO-α* was significantly decreased in mice treated with AZ8154 vs vehicle group (data not shown). However, the circulating levels of the proinflammatory cytokines GRO-α and TNF-α were reduced in mice treated with AZ8154 vs vehicle (Fig. 4c). An increase in the survival factor HGF was observed in the grafted islets in the AZ8154-treated mice (Fig. 4d). Furthermore, we also found a significant increase (50%) in the mRNA level of the beta cell-specific transcription factor *MAFA* (*p* < 0.05) (Fig. 4f) but not *PDX-1* (Fig. 4e) in islet grafts following treatment with AZ8154 vs vehicle.

### GPR44 inhibition mediates upregulation of PDX-1 via phosphorylation of Akt, GSK3β and FOXO1 in human islets

**Fig. 5:**
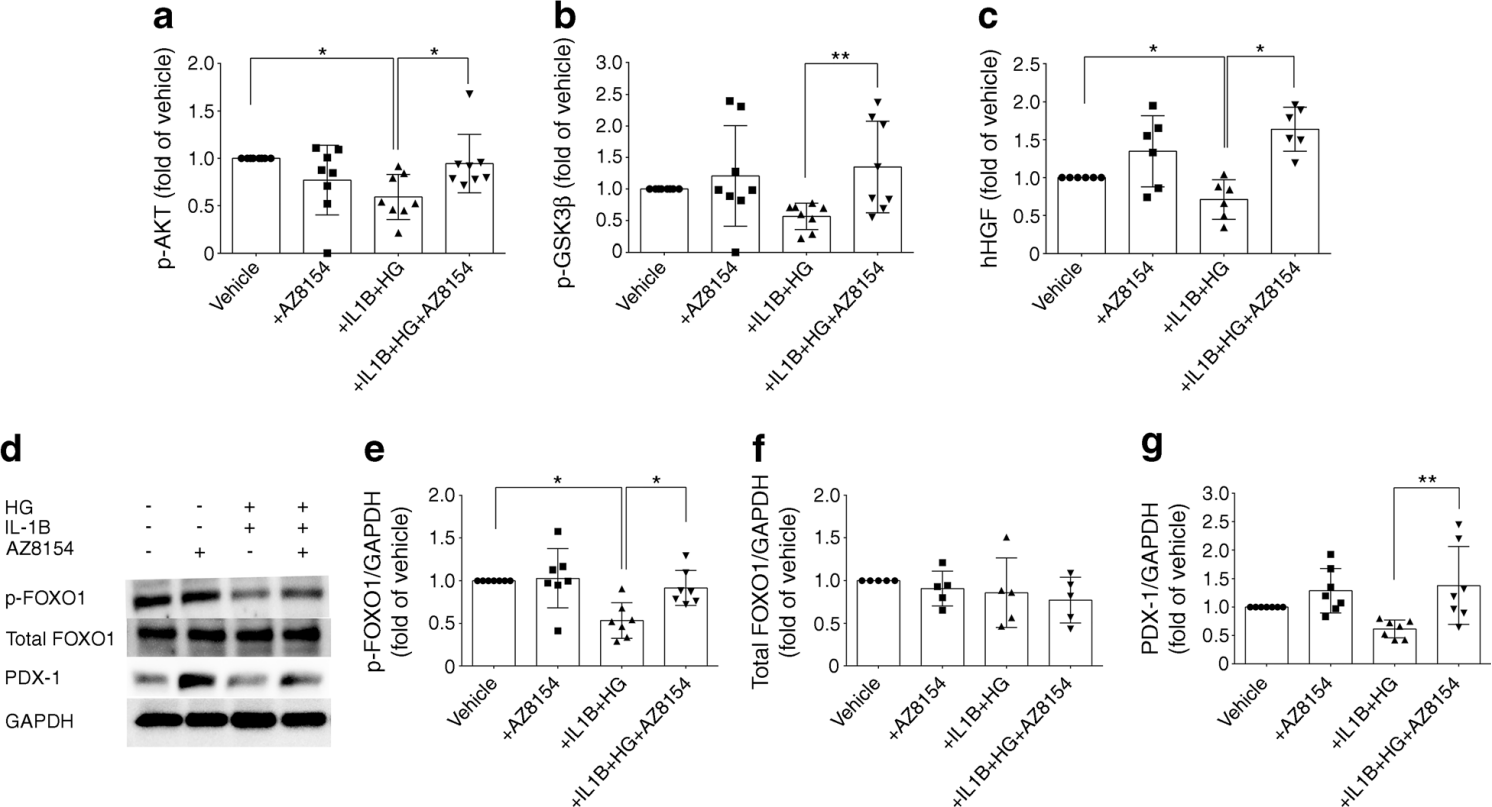
GPR44 inhibition increases protein levels of PDX-1 via the Akt–GSK3β–FOXO1 signalling pathway in isolated human islets. (**a**–**c**) Phosphorylation of Akt (**a**; *n* = 8 independent donors) and GSK3β (**b**; *n* = 8 independent donors), and secretion of HGF (**c**; *n* = 6 independent donors) in human islets treated with either AZ8154 (10 μmol/l) alone or HG (20 mmol/l) + IL-1β (10 ng/ml) with or without AZ8154 (10 μmol/l) for 48 h. (**d**) Western blot analysis of phosphorylated FOXO1, total FOXO1 and PDX-1 in human islets treated with either AZ8154 (10 μmol/l) alone or HG (20 mmol/l) + IL-1β (10 ng/ml) with or without AZ8154 (10 μmol/l) for 48 h. (**e**–**g**) Quantification of blots shown in (**d**): p-FOXO1 (**e**; *n* = 7 independent donors), total FOXO1 (**f**; *n* = 5 independent donors) and PDX-1 (**g**; *n* = 7 independent donors). Band densities were normalised to GAPDH and presented as fold of changes over control (vehicle-treated) islets. For all analyses, data were analysed with one-way ANOVA with Bonferroni corrections and presented as mean ± SD. *p < 0.05 and **p < 0.01. hHGF, human HGF

**Fig. 6:**
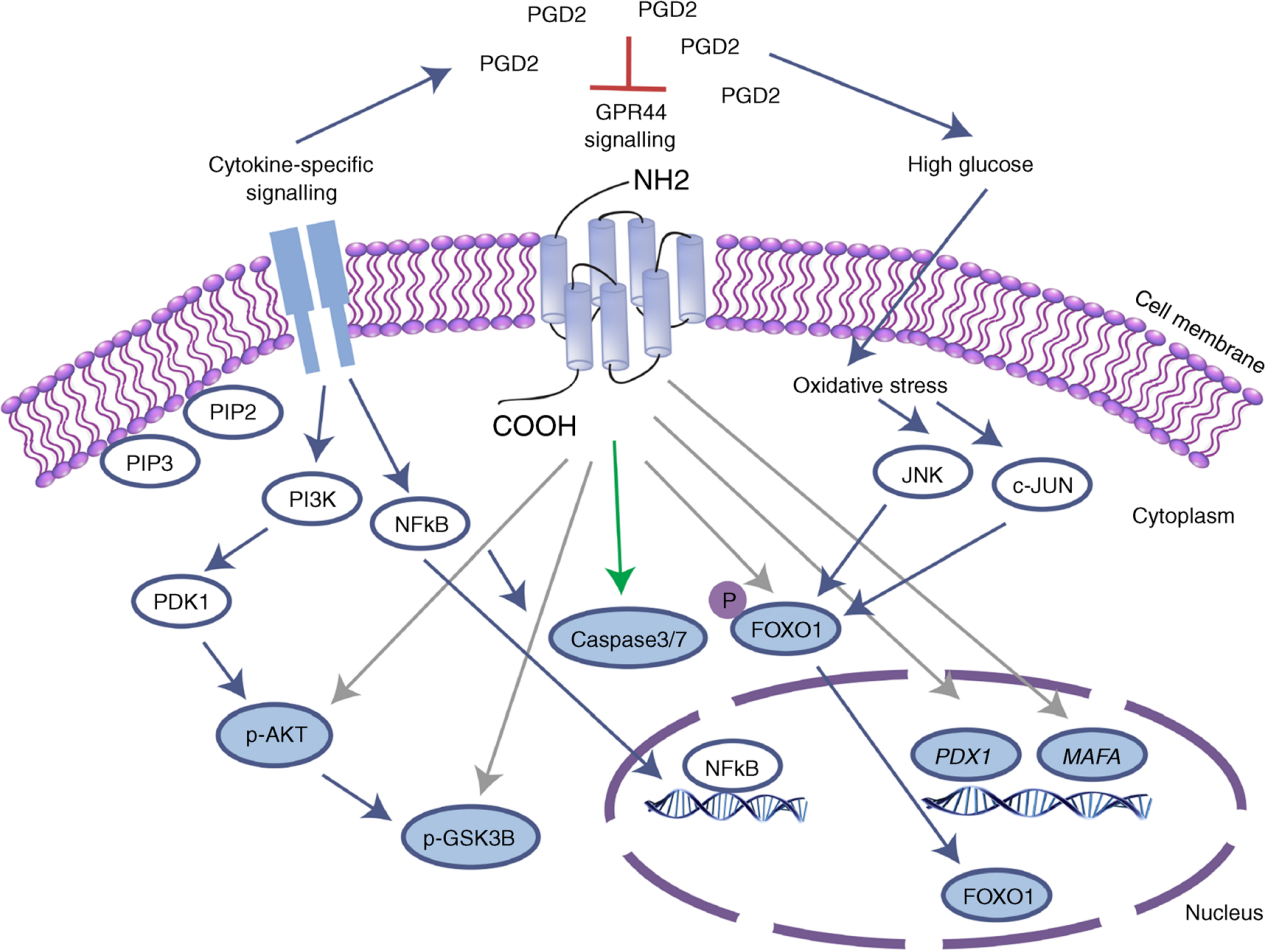
Schematic overview of the PGD_2_–GPR44 axis in human islets exposed to hyperglycaemia and proinflammatory cytokines. HG and presence of proinflammatory cytokines increases the level of PGD_2_ in the vicinity of islets [8, 9]. PGD_2_ signals through GPR44 and induces apoptosis in human islets via increase in caspase 3/7 activity (green arrow). Inhibition of the PGD_2_–GPR44 axis improves human islet viability and function via increased activation of the Akt–GSK3β signalling pathway. Inhibition of GPR44 during stress responses restores phosphorylation of FOXO1 and upregulates the expression of beta cell transcription factors, PDX-1 and MafA (grey arrows). It has been suggested that unphosphorylated FOXO1 translocates to the nucleus where it binds to *PDX-1* gene and downregulates its transcription [37, 39]. Therefore, increased phosphorylation of FOXO1 could, at least partly, be involved in the upregulation of key beta cell transcription factors

In vitro exposure of human islets to HG + IL-1β resulted in a twofold reduction in phosphorylation of Akt (Fig. 5a) and GSK3β (Fig. 5b) compared with exposure to vehicle. These reductions were reversed by AZ8154, which restored the phosphorylation of Akt and GSK3β to levels similar to those observed in vehicle-treated islets (Fig. 5a,b). In addition, HG + IL-1β reduced secretion of HGF by human islets and this effect was reversed by AZ8154 (Fig. 5c). Western blot analysis of human islets treated with HG + IL-1β revealed a twofold reduction in phosphorylation of FOXO1 (Fig. 5d,e) compared with the vehicle, whereas inhibition of GPR44 reversed the effect of HG + IL-1β (Fig. 5d,e). PDX-1 was slightly but not significantly reduced by HG + IL-1β, while AZ8154 increased the PDX-1 level in the islets exposed to this inflammatory environment (Fig. 5d,g). The protein level of total FOXO1 was unaffected regardless of the treatment (Fig. 5d,f). These data suggest that GPR44 inhibition protected human islet function at least partly through activation of the Akt–GSK3β pathway, phosphorylation of FOXO1 and upregulation of PDX-1 (Fig. 6).

## Discussion

Inhibition of GPR44 in human islets has the following effects: (1) reduced PGD_2_- and proinflammatory cytokine-induced apoptosis; (2) preserved islet grafts; and (3) improved glucose regulation in vivo in human islets exposed to a type 1 diabetes-like milieu. The mechanism is potentially related to reduction in inflammatory responses as plasma levels of TNF-α and GRO-α were decreased, while the cell survival marker HGF was increased in human islets exposed to a type 1 diabetes-like milieu in vitro and in vivo. The inhibition of GPR44 in human islets is involved in restoration of Akt, GSK3β and FOXO1 phosphorylation and increase in the expression of the transcription factors PDX-1 and V-Maf musculoaponeurotic fibrosarcoma oncogene homolog A (MafA). Our findings suggest an important role for the PGD_2_–GPR44 pathway during stress-induced responses in islets exposed to a hyperglycaemic and proinflammatory environment, representing a potential target to improve islet survival rate and function.

Hyperglycaemia and systemic inflammation, which are the main hallmarks of diabetes, induce production of prostaglandin molecules through upregulation of the COX2 enzyme [18]. Among the prostaglandin family, the PGE_2_ pathway has been extensively studied in pancreatic islets [5, 19]. PGE_2_ reduces glucose-stimulated insulin secretion in beta cell lines and in rodent and human islets [20, 21]. Moreover, intravenous infusion of PGE_2_ to rats decreased insulin secretion in response to glucose and, by using a transgenic mouse model overexpressing COX2 and PGE_2_ synthase-1, reduced insulin level and hyperglycaemia were demonstrated [22, 23]. PGE_2_ signals through four types of EP receptor (EP1–4); EP3 is the only G-protein coupled PGE_2_ receptor that couples to inhibitory G-proteins and reduces insulin secretion in response to glucose through reduced cAMP levels [21]. Antagonising the EP3 receptor improves insulin secretion in response to glucose in MIN6 beta cells and in rat islets [24]. EP3 inhibition also enhances insulin secretion in human islets isolated from donors with type 2 diabetes [21].

Elevated levels of PGD_2_ impair glucose-stimulated insulin secretion and reduce cAMP levels [7, 8]. Our data show that PGD_2_ increased apoptosis in human islets to a similar degree as proinflammatory cytokines. This effect was blocked by administration of the GPR44 antagonist AZ8154, which is an analogue to the clinical GPR44 antagonist drug candidate AZD1981 [12]. AZD1981 has been shown to reverse the effect of PGD_2_ on suppressing insulin secretion in human islets [8]. Administration of the antagonist AZ8154 to hyperglycaemic mice transplanted with a marginal number of human islets improved glucose response, increased plasma levels of human C-peptide and increased the insulin-positive area of the transplanted islets, which could suggest improvement in human islet grafts [25]. These effects were accompanied by an increased ratio of human C-peptide to fasting blood glucose, which has been shown to correlate with increase in beta cell area [26]. A recent clinical study explored the acute effect of GPR44 inhibition in individuals with poorly controlled type 2 diabetes [8]. The outcome was negative in terms of the administration of the GPR44 antagonist AZD1981 having no acute effect on insulin secretion. It is possible that these individuals had already developed significant islet dysfunction and therefore did not respond to acute administration of the drug, or an elevated PGD_2_ tone is not present in the islet vicinity. Moreover, considering the specific expression of the GPR44 receptor on beta cells, the presence of too few functional beta cells could be another reason for the absence of an effect of GPR44 inhibition in individuals with type 2 diabetes [1, 8].

In the islet transplantation process, it is well known that proinflammatory cytokines are involved in pancreatic islet injury and islet graft destruction post transplantation [27]. In particular, elevated levels of proinflammatory cytokines IL-1β, TNF-α and INF-γ observed after islet transplantation can mediate islet injury at the early phase post transplantation [28]. IL-1β production is triggered by itself and by high concentration of glucose within islets [29]. Furthermore, production of the proinflammatory cytokine GRO-α has been found to increase 6 h after human islet exposure to whole blood using an ex vivo loop blood model mimicking the graft loss post transplantation [30]. GPR44 is highly expressed in human islets [1, 8, 11] and we found that the inhibition of GPR44 led to reduced plasma levels of human GRO-α and TNF-α in the post islet transplantation phase. This could support the positive impact of GPR44 inhibition on reducing the adverse effects of the inflammatory responses that are often responsible for major loss of functional beta cell mass after islet transplantation [31].

Increased secretion of proinsulin could occur due to the presence of a stress micro-environment such as prolonged exposure to hyperglycaemia and elevated demand for insulin secretion [32, 33]. Increased ratio of proinsulin to insulin has been reported in individuals with new onset of type 1 diabetes and in allo-islet transplanted patients [34, 35]. It is not clear from our study the extent to which the reduced ratio of proinsulin to insulin in GPR44-treated mice contributes to the preserved functionality of transplanted islets, although the reduction in this ratio accompanied by elevated level of C-peptide and mRNA expressions of *PDX-1* and *MAFA* together with a reduction in secretion of proinflammatory cytokines could suggest an overall improvement of the human islet grafts.

PGE_2_ has been found to both decrease DNA synthesis and to reduce islet survival rate in rat islets [36]. This was suggested to be mediated through PGE_2_ coupling to the EP3 receptor, leading to dephosphorylation of FOXO1 either via activation of c-Jun N-terminal kinase (JNK1) or inhibition of the Akt survival signalling pathway [37, 38]. Subsequently there is an increase in translocation of FOXO1 to the nucleus where it could participate in nuclear exclusion of critical beta cell transcription factors, PDX-1 and MafA [37, 39]. MafA and PDX-1 have been previously suggested to be key activators of insulin synthesis and master regulators of genes involved in maintaining beta cell function [40]. We observed a reduction in protein expression of PDX-1 in human islets exposed to a diabetic milieu in vitro, and also found a decrease in phosphorylation of Akt and its downstream target GSK3β. Importantly, the effect of the stressors was reversed by inhibition of GPR44, shown by upregulation of PDX-1 protein expression, increased phosphorylation of FOXO1 and activation of the Akt–GSK3β signalling pathway. Furthermore, we demonstrated upregulation of HGF in islet grafts in the early phase post transplantation and in human islets exposed to a type 1 diabetes-like milieu and treated with GPR44 antagonist in vitro. HGF has been shown to be involved in regeneration and restoration of damaged tissue including pancreatic beta cells [41]. Proinflammatory cytokines have been reported to modulate secretion of HGF in human islets in vitro [42]. Adenoviral delivery of HGF to islets from non-human primates demonstrated a marked improvement in transplanted islet function and mass suggesting a possible role for HGF in the survival of islets exposed to diabetogenic agents [43]. HGF is also known for improving insulin secretion, mainly through activation of the Akt signalling pathway [44]. Therefore, HGF could at least partly be involved in activation of the Akt signalling pathway and improvement in islet function. Despite similar adverse effects of PGD_2_ and PGE_2_ on human islets, and similar signalling pathways, the positive effects we observed with AZ8154 was only related to GPR44 since the compound had no activity on the PGE_2_ receptor EP3.

This study supports a role for the PGD_2_–GPR44 pathway in human islet dysfunction and suggests that the inhibition of GPR44 could improve islet survival rate and function during severe hyperglycaemia. Inhibition of GPR44 may have the potential to represent a rescue strategy for islets exposed to an acute inflammatory and hyperglycaemic environment and may preserve human islet grafts after transplantation.

## Electronic supplementary material

ESM (PDF 616 kb)

## Data Availability

The datasets generated during and/or analysed during the current study and presented in the main article and as supplementary materials are available from the corresponding authors upon a reasonable request. The chemical structure of AZ8154 compound, which was generated by Astra Zeneca and used in the current study, is not publicly available due to the fact that the compound has not been patented yet but is available from the corresponding author upon reasonable request.

## Abbreviations

CXCL1: Chemokine (C-X-C motif) ligand 1
EIA: Enzyme immunoassay
FOXO1: Forkhead box O-1
GRO-α: Growth-regulated oncogene-α
GSK3β: Glycogen synthase kinase 3β
HG: High concentration of glucose
HGF: Hepatocyte growth factor
HPMC: Hydroxypropylmethylcellulose
MafA: V-Maf musculoaponeurotic fibrosarcoma oncogene homolog A
PDX-1: Pancreatic and duodenal homeobox-1
PGD_2_: Prostaglandin D_2_
PGE_2_: Prostaglandin E_2_
PI3K: Phosphoinositide 3-kinases
PIP: Phosphatidylinositol
