# Supplementary material for: Inhibition of the prostaglandin D2–GPR44/DP2 axis improves human islet survival and function
Source: Diabetologia. 2020 Apr 29;63(7):1355–67. doi: 10.1007/s00125-020-05138-z (PMC7286861; doi:10.1007/s00125-020-05138-z)
Supplement: Supplementary file 1 — (PDF 616 kb) [file 125_2020_5138_MOESM1_ESM.pdf]

Abadpour S et al. Inhibition of the prostaglandin D2-GPR44/DP2 axis improves human islet survival and function

Electronic Supplementary Materials (ESM)

ESM Methods

Compounds and in vitro pharmacology

Determination of GPR44 antagonist potency by scintillation proximity assay

Recombinant human GPR44, human DP1 and mouse GPR44 were expressed in the human embryonic kidney 293 cell line (HEK293 cells) and used in the binding assay. Membrane preparations were stored in aliquots at -80°C. Binding of [^{3}H]-PGD_2 (Perkin Elmer/NEN) and specific compounds were assessed by a scintillation proximity assay (SPA) after 2 h at room temperature in the presence of 1 % (vol/vol) DMSO. Concentration-dependent inhibition of [^{3}H]-PGD_2 binding by the compounds was expressed as IC_{50}.

The assay was performed in white 96 well clear-bottomed NBS plates (Corning). Prior to the assay, the HEK cell membranes expressing GPR44 or DP1 were coated onto SPA PVT WGA beads (Amersham) and were re-suspended in membrane buffer to give 100 mg beads/ml. For coating, membranes were incubated with beads at 4°C with constant agitation. The beads were pelleted by centrifugation (800 x g for 10 minutes at 4°C), washed once with assay buffer (50 mmol/l HEPES pH 7.4 containing 5 mmol/l $MgCl_2$) and finally re-suspended in assay buffer at a bead concentration of 10 mg beads/ml.

5 μl test compound (or assay buffer containing 10% DMSO for determination of total binding) was added to each well, followed by 20 μl of 6.25 nmol/l [^{3}H]-PGD_2 and 25 μl of membrane coated beads prepared as described above. Non-specific binding was determined by adding 5 μl of 100 μmol/l 13,14-dihydro-15-keto PGD_2 (Cayman Chemicals) in place of test compound. After 2 h incubation at room temperature, the plate was read on a Wallac MicroBeta counter using a ^{3}H protocol for 4 minutes per well.

Specific binding was determined from controls performed in each assay plate. The non-specifically bound radioactivity was subtracted from each well. Data were expressed as percent inhibition of specific $[^3H]$-PGD_2 binding and IC_{50} was determined for each compound.

Glucose-stimulated insulin secretion assay with EndoC-βH1 cells

Human EndoC-βH1 cells (Univercell Biosolutions, Toulouse, France [mycoplasma negative]) [1] were plated in fibronectin/extracellular matrix-coated 96-well plates at a density of 5×10^4 cells per well and cultured in maintenance media for 48 h at 37°C, 5 % CO_2 followed by replacing the maintenance medium containing low glucose (2.8 mmol/l) for 16 h. Glucose-stimulated insulin secretion (GSIS) experiments were performed at 37°C, 5 % CO_2 by incubating the cells in KREBS buffer containing 0.2 % wt/vol BSA, 0.5 mmol/l 3-isobutyl-1-methyl-xanthine (IBMX, a phosphodiesterase inhibitor) with either 2.8 (LG) or 11.1 (HG) mmol/l glucose, in the presence of serial dilutions of the GPR44 antagonists AZ8154 and AZD1981 at a concentration range of 3.81×10^{-11} mol/l to 2.5×10^{-6} mol/l, with or without EC_{80} (150 pmol/l) of the GPR44 agonist 15(R)-15-methyl-prostaglandin(PG) D_2 [2]. The effect of AZ8154 and AZD1981 inhibitors on insulin secretion was determined at 11.1 (HG) mmol/l glucose with 150 pmol/l 15(R)-15-methyl-PGD_2. At the end of the incubation period (30 min), supernatants were collected, and insulin levels were determined by an HTRF®-based insulin assay according to the manufacturer´s instructions (Cisbio Bioassays).

cAMP measurement assay with EndoC-βH1 cells

Human EndoC-βH1 cells were plated as described above and pre-incubated for 1 h in KREBS buffer with 0.2% wt/vol BSA and 0.5 mmol/l glucose at 37°C, 5% CO_2. cAMP experiments were performed with 30 minutes incubation of the cells at 37°C, 5% CO_2 in KREBS buffer containing 0.2% wt/vol BSA, 0.5 mmol/l 3-isobutyl-1-methyl-xanthine (IBMX, a phosphodiesterase inhibitor) and either 2.8 (LG) or 11.1 (HG) mmol/l glucose, in the presence

of serial dilutions of AZ8154 and AZD1981 at a concentration range of 3.81×10^{-11} mol/l to 2.5 $\times10^{-6}$ mol/l, with or without EC_{80} (150 pmol/l) of the GPR44 agonist 15(R)-15-methyl-PGD_2. At the end of the incubation period, cells were lysed, and cAMP levels in the cell lysate were measured using an HTRF®-based cAMP assay according to the manufacturer´s instructions (Cisbio Bioassays).

Selectivity assay of AZD1981 and AZ8154 on EP3

The PGE_2 receptor, prostaglandin E receptor 3 (EP3) is highly expressed in human adipocytes [3]. A label-free assay was set up in human adipocytes to test EP3 activity of the GPR44 antagonists, ADZ1981 and AZ8154. The Epic® label-free screening system (Corning®) measures the dynamic mass redistribution (DMR) of cells, which enables real-time detection of integrated cellular responses in living cells. The method relies on detection of refractive index alterations on biosensor-coated microplates that originate from changes in the total biomass proximal to the sensor surface in response to a stimulus such as activation of a G-protein coupled receptor.

Human pre-adipocytes were isolated from human adipose tissue and cryopreserved. Initially, cells are thawed, seeded in 384-well fibronectin-coated EPIC™ biosensor plates (Corning), after which differentiation is initiated by addition of an adipogenic cocktail (1 µmol/l Dexamethasone (Sigma), 0.1 µmol/l Insulin (Actrapid, NovoNordisk, Denmark), 1 µmol/l Pioglitazone, and 50 µmol/l IBMX (Sigma)). After 2 weeks of differentiation, the cells have visible lipid droplets and express adipocyte-specific markers. On the day of the experiment, cells were washed with buffer (1xHBSS, 20 mmol/l HEPES (pH 7.4) and 0.01% wt/vol BSA) and allowed to equilibrate for 1 h inside the EPIC™ plate reader (Corning) at 26°C. Following equilibration, a 5 min scan was performed to create a baseline read before applying the EP3 agonist, Sulprostone [4] in different concentrations diluted in buffer (1xHBSS, 20 mmol/l

HEPES (pH 7.4) and 0.01 % BSA) using a CyBi-Well vario (CyBio) to determine the EC_{80} concentration. The selectivity of the AZD1981 and AZ8154 was determined at EC_{80} concentration of Sulprostone. The real-time measurement of DMR was detected during a 60 min scan.

Pharmacokinetic analysis of AZ8154 and AZD1981

Prior to the experiments, the mice and rats were maintained in a temperature-controlled room (22 ^{o}C) on a 12 h light-dark cycle and were allowed to acclimatize for one week after arrival. The animals were housed in plastic cages with wooden bedding and nesting material in groups of five individuals with free access to lab chow (R70, Lantmännen, Sweden) and tap water. Principles of laboratory animal care were implemented according to Swedish authorities, and the studies were approved by the Animal Ethics Committee in Gothenburg, Sweden.

To determine the dose of AZ8154 for administration to diabetic mice transplanted with human islets, a pharmacokinetic experiment was performed in female C57BL6/JOlaHsd mice (Harlan, Germany). Mice were dosed orally with three different doses (3, 30 and 150 mg/kg, respectively) of AZ8154 formulated in 0.5 % wt/vol hydroxypropyl methyl cellulose (HPMC). Blood samples were taken from the tail vein 0.5, 1, 2, 4, 8 and 24 h after administration of the compound. Concentration of the compound in the blood was analyzed using reversed-phase high-pressure liquid chromatography with rapid gradient elution followed by mass spectrometry detection. The detection limit was set to 75 nmol/l.

In a separate study in Wistar rats (Harlan, Germany), AZD1981 and AZ8154 were orally administered with a single dose of 3, 30 or 150 mg/kg, and the pharmacokinetic profile of the compounds over 24 h was measured as described above.

Cell death and apoptosis analysis with human islets

Programmed cell death was analyzed in human islets by detection of DNA-histone complexes in the cytoplasmic fraction of islet lysates using the Cell Death Detection ELISA^{PLUS} kit (Roche Diagnostics, Mannheim, Germany) according to the manufacturer´s protocol.

The level of caspase activity in human islet cells was determined by the Caspase-Glo 3/7 assay according to the manufacturer´s protocol (Promega Biotech, Madison, WI, USA).

Glucose-stimulated insulin secretion assay with human islets

Twenty equally-sized human islets were handpicked and transferred into a transwell plate (Corning, NY, USA) containing Krebs-Ringer bicarbonate buffer (11.5 mmol/l $NaCl_2$, 0.5 mmol/l KCl, 2.4 mmol/l $NaHCO_3$, 2.2 mmol/l $CaCl_2$, 1 mmol/l $MgCl_2$, 20 mmol/l HEPES, and 2 mg/l albumin: all Sigma-Aldrich) supplemented with 1.67 mmol/l glucose and incubated for 45 min at 37 °C. Transwells were switched to Krebs-Ringer bicarbonate buffer containing 20 mmol/l glucose and incubated for 45 min at 37 °C. Supernatants were harvested for analysis of insulin secretion using a human insulin ELISA kit (Mercodia AB, Uppsala, Sweden). Stimulation index (SI) was calculated as a ratio of insulin secreted at high glucose (20 mmol/l) versus low glucose (1.67 mmol/l).

Glucose measurements and IVGTT

Random non-fasting blood glucose and body weight were monitored every other day in the morning until termination of the study. The islet response to glucose was investigated by IVGTT on day 10, 4 h after administration of the GPR44 antagonist to the animals. Mice were fasted for 4 h before an intravenous D-glucose injection (1 g/kg body weight, Fresenius Kabi, Bad Homburg, Germany) via a tail vein. Blood glucose was measured before (0), and 1, 5, 15, 45 min after glucose administration using a glucometer (Accu-Chek Aviva Nano, Roche

Diagnostics, IN, USA). To exclude acute effects of the GPR44 antagonist, a second IVGTT was performed on day 15 without the morning administration of the GPR44 antagonist, when exposure to the compound was expected to be low according to the pharmacokinetic profile of the compound (ESM Fig 3).

Biochemical measurements

In all in vivo studies, blood samples were harvested from 4 h fasted mice on day 2 and 10 post transplantation from the saphenous vein and at termination by heart puncture after euthanizing the animals. Blood samples were collected into EDTA-coated or serum tubes (Becton, Dickinson and Company, Franklin Lakes, NJ, USA).

Assessment of human specific C-peptide, proinsulin and insulin in plasma samples were performed by using EIA assays (Mercodia) according to the manufacturer´s protocol. Levels of cytokines, TNF-α and GRO-α were measured in plasma samples using U-PLEX electrochemiluminescence immunoassay on MESO Quick analyzer (Meso Scale Diagnostics, MD, USA). The protein level of HGF, phosphorylated AKT and GSK3β were measured in the human islet lysate utilizing multiplex immunoassay with magnetic beads and detected with a multiplex analyzer (Biorad, CA, USA) according to the manufacturer´s instructions.

Immunofluorescent Staining

At termination of in vivo studies, the entire islet grafts were harvested and fixed in 10% formalin and embedded in paraffin followed by sectioning of the grafts every 25 µm with slide thickness of 8 µm. Islet grafts were stained with guinea pig anti-insulin polyclonal antibody 1:500 (DAKO, Oslo, Norway). Donkey-anti-guinea pig Alexafluor 594 1:300 (Life Technologies AS, Oslo, Norway) was used as secondary antibody to detect expression of insulin followed by nuclear staining using SlowFade Gold antifade reagent with DAPI (Life Technologies AS, Oslo, Norway) in transplanted islets. Slides were scanned and images were taken by the slide

scanner Axio scan Z1 (Carl Ziess AS, Germany) operated by the ZEN lite blue software. The area of insulin-positive cells was analyzed using Image J software (National Institute for Health, USA).

Real-time qPCR

Frozen harvested islet grafts were homogenized, and total RNA was isolated using TRIrizol®/Chloroform (Qiagen, CA, USA) for phase separation followed by the RNeasy Micro Kit (Qiagen, Hilden, Germany) according to the manufacturer´s guidelines. The concentration of total RNA samples was measured by NanoDrop ND-1000 UV/Vis spectrophotometer (Saveen Werner AB, Sweden). Following cDNA synthesis using the High-Capacity cDNA Archive Kit (Applied Biosystems, Foster City, CA, USA), mRNA expression was analyzed using the following TaqMan primers and probes: human v-maf musculoaponeurotic fibrosarcoma oncogene family, protein A (*MAFA*) Hs01651425_s1, human pancreatic and duodenal homeobox 1 (*PDX1*) Hs00426216_m1, Tumor Necrosis Factor (TNF-α) Hs00174128_m1 and C-X-C motif chemokine ligand 1/GRO-α (GRO- α) Hs00236937_m1with an ABI 7900HT standard Real-Time PCR System (Applied Biosystems, CA, USA). Results were normalized to the housekeeping gene human *RPL-30* (Hs00265497_m1). All primers were provided from Life Technologies AS, Oslo, Norway.

Western Blot Analysis

Cell lysis buffer (RIPA buffer supplemented with Halt protease inhibitor, Thermo scientific, Oslo, Norway) was added to human islets before proceeding to mechanical disruption using sonication. Samples were centrifuged and purified using a QIAshredder purification column (QIAGEN, Hilden, Germany). Total protein concentration was determined using the Pierce BCA protein assay (Life Technologies AS, Oslo, Norway). Equal amount of total protein (20 μg) were analyzed by immunoblotting with rabbit primary antibodies against phosphorylated

FOXO1 or total FOXO1 (Cell Signaling, MA, USA), PDX1 (Abcam, Cambridge, UK), or a goat polyclonal Glyceraldehyde-3-Phosphate Dehydrogenase (GAPDH) antibody (Santa Cruz Biotechnology, TX, USA), all at the concentration of 1:1000. Bound antibodies were labeled with goat anti-rabbit IgG-HRP and donkey anti-goat IgG-HRP (Santa Cruz Biotechnology, TX, USA) 1:10000. Protein bands were visualized using the Clarity western ECL chemiluminescence substrate kit (Biorad, CA, USA) or the Super signal west femto reagents (Thermo scientific, Oslo, Norway) followed by semi-quantitative measurements of band density using the chemiDGC touch imaging system, (BioRad, CA, USA).

ESM Results

AZ8154 and AZD1981 were equally potent in blocking PGD_2 signaling in human beta cells

The potency of the GPR44 antagonists was determined using a scintillation proximity assay in which concentration dependent inhibition of $[^3H]$-PGD_2 binding by compounds was expressed as IC_{50}. AZ8154 was found to be slightly more potent on human GPR44 compared to mouse GPR44 (ESM Table 1), and both compounds were >1000-fold more potent on human GPR44 than on human DP1 receptor (ESM Table 1). The in vitro pharmacological properties of AZ8154 and AZD1981 were studied in the human beta cell line EndoC-βH1 using cAMP measurement and insulin secretion. Glucose (11.1 mmol/l) stimulated insulin secretion in the EndoC-βH cells about 2-fold over basal secretion (2.8 mmol/l glucose) and this effect was blocked by addition 150 pmol/l 15(R)-15-methyl-PGD_2 (ESM Fig 1a). Addition of 100 nmol/l AZD1981 or 100 nmol/l AZ8154 reversed the inhibitory effect of PGD_2 and restored the GSIS response. The same pattern was observed during cAMP measurement, with a significant inhibition of cAMP production by PGD_2 and complete restoration to stimulated levels by both AZD1981 and AZ8154 (ESM Fig. 1b).

The dose response curves for the two GPR44 antagonists were compared and the IC_{50} values were found to be similar between the two compounds with similar effects on GSIS (ESM Fig. 1c) or cAMP production (ESM Fig. 1d). The potencies of the compounds were measured multiple times and the results are summarized in ESM Table 2. In conclusion, both GPR44 antagonists, AZ8154 and AZD1981, demonstrated similar potencies in antagonizing PGD_2-induced inhibition of cAMP production and GSIS in EndoC-βH1 cells.

EP3 selectivity for AZD1981 and AZ8154

EP3 selectivity for AZD1981 and AZ8154 was determined using a label-free EPIC assay. The selective EP3 agonist, Sulprostone, was used to demonstrate EP3 activity in the primary human adipocytes, by adding 10^{-5} to $10^{-9.5}$ mol/l of the agonist. A dose response curve was established and EC_{50} for Sulprostone was determined to 47 nmol/l. The effect of AZD1981 and AZ8154 on EP3 activity were measured at EC_{80} (180 nmol/l) of Sulprostone. There was no activity at any of the concentrations of the GPR44 antagonists on EP3 signaling in human primary adipocytes (ESM Fig 2).

Pharmacokinetic evaluation of AZ8154 for determination of dose in the human islet transplantation studies

While AZD1981 and AZ8154 were equally potent in vitro in restoring GSIS and cAMP production (ESM Fig. 1), AZ8154 demonstarted better in vivo pharmacokinetic profile with longer half-life (6.2 h vs. 3.7 h) and 10-fold difference in the 24 h plasma exposure (ESM Fig. 3a-b). This resulted in a lower dose of AZ8154 compared to AZD1981 to achieve plasma concentrations of the compound that were expected to fully inhibit GPR44. Therefore, the pharmacodynamics assessment of GPR44 inhibition on human islet function in vivo was explored using AZ8154.

The initial pharmacokinetic study with three dose groups and dense sampling indicated that the dose proportional exposure increases between the low dose (3 mg/kg; AUCu=110 nmol/l$\times$ h) and the middle dose (30 mg/kg; AUCu=1900 nmol/l$\times$h) (ESM Fig. 4a). The exposure of the high dose (150 mg/kg; AUC=11000 nmol/l $\times$ h) was roughly dose proportional to the exposure of the middle dose. The dose level of 50 mg/kg twice daily in the pharmacodynamic study was chosen to achieve an unbound average exposure about 25 times greater than the in vitro IC_{50} in the GSIS and cAMP assays, and to achieve full inhibition over 24 h (ESM Fig. 4b). Observed

exposure in the human islet transplanted mice was more than 2-fold of the predicted exposure level (predicted unbound plasma concentration at 4 h was 360 nmol/l, and the corresponding observed value was 800±240 nmol/l). The pharmacodynamic study were performed to set the following rationale: the low dose of 3 mg/kg twice daily was chosen to achieve an unbound average exposure at the level of in vitro IC_{50} in the insulin and cAMP assays, and the high dose of 30 mg/kg twice daily was chosen to achieve full inhibition over 24 h (ESM Fig. 4c). For the high dose group, the predicted exposure was achieved (predicted unbound plasma concentration at 4 h was 210 nmol/l, and the corresponding observed value was 150±83 nmol/l). However, the low-dose group only reached about 40 % of the predicted exposure level (predicted unbound plasma concentration at 4 h was 11 nmol/l, and the corresponding observed value was 4.0±2.4 nmol/l) (ESM Fig. 4c).

ESM tables:

ESM Table 1: Binding potencies for AZ8154 and AZD1981 on human and mouse GPR44 and human DP1

	**pIC_{50} AZ8154 (mean±SD)**	**pIC_{50} AZD1981 (mean±SD)**
Human GPR44	8.4±0.1 (n=2)	8.4±0.3 (n=25)
Mouse GPR44	7.9±0.1 (n=2)	8.3±0.1 (n=2)
Human DP1	<5.0 (n=2)	<5.0 (n=2)

The binding assay was performed in at least two independent experiments for each compound using HEK293 cells overexpressing human or mouse GPR44, or human DP1. The results are expressed as pIC_{50} values (negative logarithmic value of the IC_{50} value).

ESM Table 2 Potencies of AZ8154 and AZD1981 on inhibition of cellular responses mediated by GPR44 in human EndoC-βH1 cells

**Assay readout**	**IC_{50} AZ8154 (nmol/l) (mean±SD)**	**IC_{50} AZD1981 (nmol/l) (mean±SD)**
cAMP	16±3.6 (n=4)	21±9.1 (n=2)
Insulin secretion	21±8.7 (n=3)	24±19 (n=3)

Both assays were run with 8 replicates of each compound concentration and the assays were repeated 2-4 times as indicated in the table.

ESM figures:

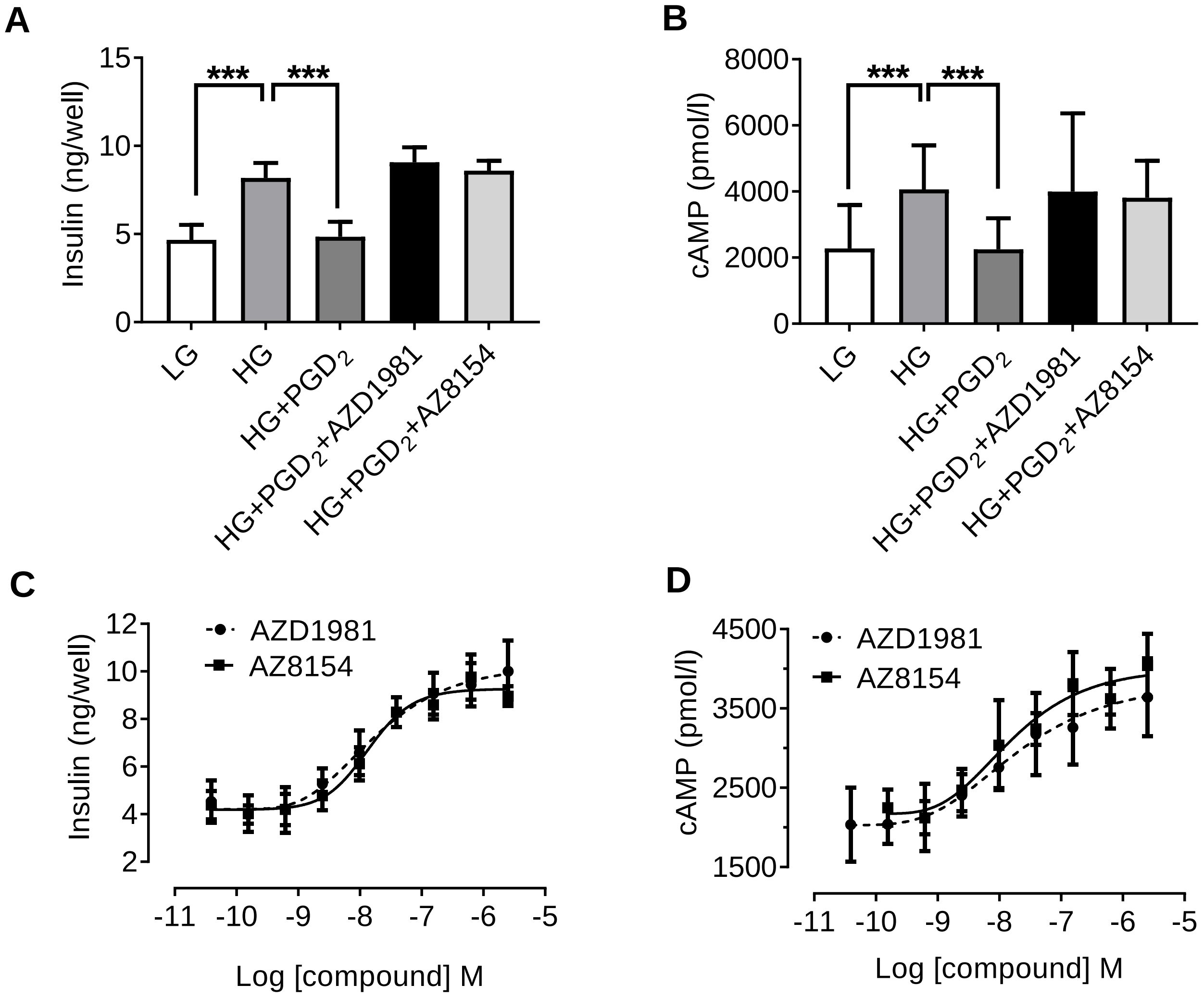

ESM Fig. 1 The level of (A) secreted insulin and (B) intracellular cAMP measured in the human beta cell line EndoC-βH1 treated with LG, HG or HG+PGD_2 with or without AZD1981 or AZ8154. In vitro potencies of the GPR44 antagonists AZD1981 and AZ8154 were determined by measuring insulin secretion (C) and intracellular cAMP (D) in EndoC-βH1 cells. Data are presented as means ± SD and analyzed by one-way ANOVA with Bonferroni corrections ***$p<0.001$. LG: Low Glucose, HG: High Glucose.

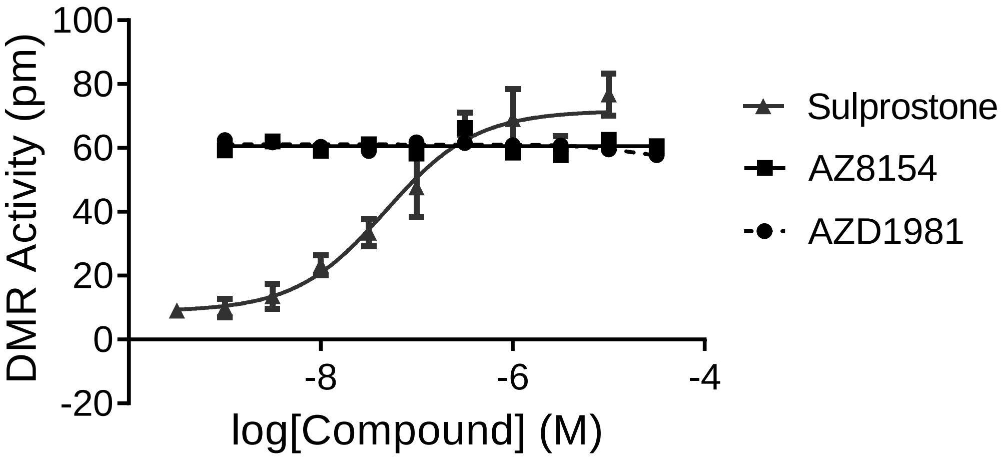

ESM Fig. 2 EP3 selectivity for AZD1981 and AZ8154 was determined using a label-free Epic assay in human primary adipocytes. Sulprostone, an EP3 agonist, was added at EC_{80} concentration when determining the selectivity for AZD1981 and AZ8154. Each concentration was analyzed in duplicates.

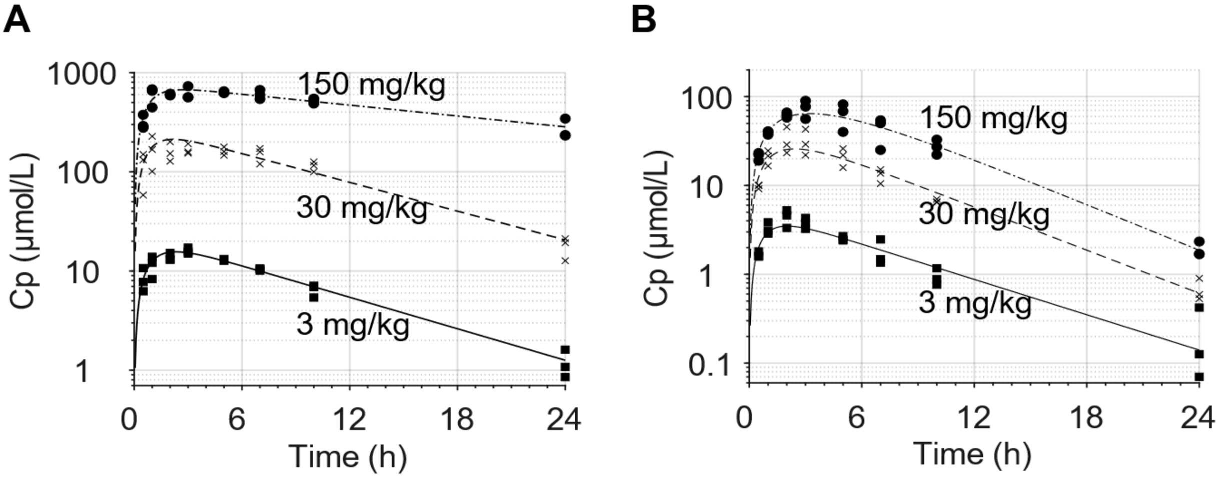

ESM Fig. 3 Plasma pharmacokinetic profiles of (A) AZ8154 and (B) AZD1981 in healthy, non-diabetic rats administered with 3, 30 or 150 mg/kg in a single dose (n=3 per group).

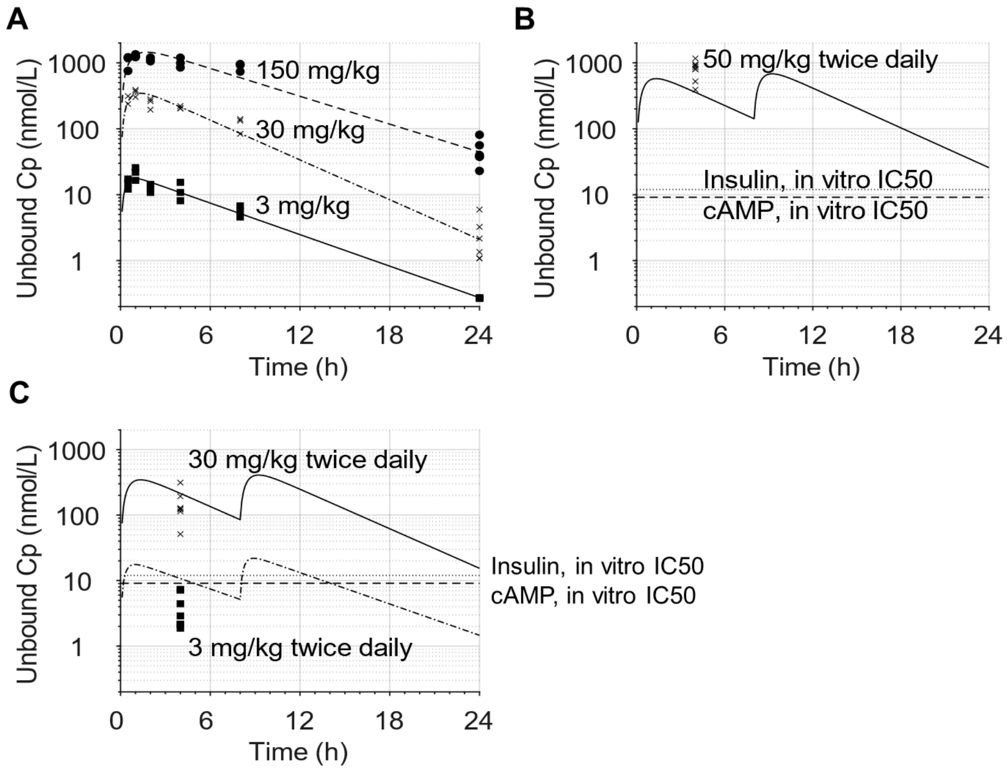

ESM Fig. 4 (A) Unbound plasma pharmacokinetic profiles of AZ8154 with the doses 150 mg/kg (dashed line modelled, circles observed), 30 mg/kg (dash-dotted line modelled, crosses observed) and 3 mg/kg (solid line modelled, squares observed) in healthy, non-diabetic mice. Unbound plasma concentration of (B) AZ8154 50 mg/kg twice daily (solid line predicted, crosses observed) and (C) 30 mg/kg twice daily (solid line predicted, crosses observed) and 3 mg/kg twice daily (dash-dotted line predicted, squares observed) in the diabetic mice transplanted with human islets sampled 17 days post transplantation. In vitro IC_{50} for the insulin (dotted line) and the cAMP (dashed line) assays are indicated in B and C. Blood samples were collected at termination of the different studies.

References:

[1] Ravassard P, Hazhouz Y, Pechberty S, et al. (2011) A genetically engineered human pancreatic beta cell line exhibiting glucose-inducible insulin secretion. Journal of Clinical Investigation 121(9): 3589-3597. 10.1172/JCI58447

[2] Skrtic S, Tyrberg B, Broberg M, et al. (2018) Exploring the insulin secretory properties of the PGD2-GPR44/DP2 axis in vitro and in a randomized phase-1 trial of type 2 diabetes patients. PloS one 13(12): e0208998. 10.1371/journal.pone.0208998

[3] Michaud A, Lacroix-Pepin N, Pelletier M, et al. (2014) Expression of genes related to prostaglandin synthesis or signaling in human subcutaneous and omental adipose tissue: depot differences and modulation by adipogenesis. Mediators of inflammation 2014: 451620. 10.1155/2014/451620

[4] Xu H, Fu JL, Miao YF, et al. (2016) Prostaglandin E2 receptor EP3 regulates both adipogenesis and lipolysis in mouse white adipose tissue. J Mol Cell Biol 8(6): 518-529. 10.1093/jmcb/mjw035

Checklist for reporting human islet preparations used in research

Adapted from Hart NJ, Powers AC (2018) Progress, challenges, and suggestions for using human islets to understand islet biology and human diabetes. Diabetologia https://doi.org/10.1007/s00125-018-4772-2

Islet preparation	1	2	3	4	5	6	7	8[a]
MANDATORY INFORMATION								
Unique identifier	O51	O59	O60	O26	O22	H2259	H2298	H2309
Donor age (years)	52	45	54	53	35	67	64	54
Donor sex (M/F)	F	F	F	F	M	M	F	M
Donor BMI (kg/m^2)	26.6	26.0	29.4	30.5	23.5	39.0	N/A	42.6
Donor HbA_{1c} or other measure of blood glucose control	5.4 (36)	N/A	N/A	4.2 (22)	N/A	4.4 (24.3)	5.3 (34)	5.5 (37)
Origin/source of islets[b]	ECIT	ECIT	ECIT	ECIT	ECIT	ECIT	ECIT	ECIT
Islet isolation centre	Oslo	Oslo	Oslo	Oslo	Oslo	Uppsala	Uppsala	Uppsala
Donor history of diabetes? Please select yes/no from drop down list	No	No	No	No	No	No	No	No
If Yes, complete the next two lines if this information is available								
Diabetes duration (years)								
Glucose-lowering therapy at time of death[c]								
RECOMMENDED INFORMATION								
Donor cause of death	DCD	DBD	DBD	DBD	DBD	DBD	DBD	DBD
Warm ischaemia time (h)	00:23	-	-	-	-	-	-	-
Cold ischaemia time (h)	3:19	5:24	9:16	4:29	5:23	9:08	8:48	15:29
Estimated purity (%)	90	75	80	80	95	60	55	75
Estimated viability (%)	95	80	95	85	95	97	90	85
Total culture time (h)[d]	48	48	48	48	48	72	72	72
Glucose-stimulated insulin secretion or other functional measurement[e]	2.1	2.3	3.4	3.9	3.2	2.4	2.07	2.7
Handpicked to purity? Please select yes/no from drop down list	Yes	Yes	Yes	Yes	Yes	Yes	Yes	Yes

Additional notes								

Islet preparation	1	2	3	4	5	6	7	8[a]
MANDATORY INFORMATION								
Unique identifier	H2310	H2167						
Donor age (years)	67	69						
Donor sex (M/F)	F	F						
Donor BMI (kg/m^2)	25.5	19.1						
Donor HbA_{1c} or other measure of blood glucose control	N/A	N/A						
Origin/source of islets[b]	ECIT	ECIT						
Islet isolation centre	Uppsala	Uppsala						
Donor history of diabetes? Please select yes/no from drop down list	No	No						
If Yes, complete the next two lines if this information is available								
Diabetes duration (years)								
Glucose-lowering therapy at time of death[c]								
RECOMMENDED INFORMATION								
Donor cause of death	DBD	DBD						
Warm ischaemia time (h)	-	-						
Cold ischaemia time (h)	6:23	9:40						
Estimated purity (%)	65	55						
Estimated viability (%)	90	90						
Total culture time (h)[d]	72	72						
Glucose-stimulated insulin secretion or other functional measurement[e]	2.5	3.8						
Handpicked to purity? Please select yes/no from drop down list	Yes	Yes						

Additional notes								

Islet preparation	1	2	3	4	5	6	7	8[a]
MANDATORY INFORMATION								
Unique identifier	HP-14289	HP-13310	HP-13318	HP-13282				
Donor age (years)	39	36	29	52				
Donor sex (M/F)	F	M	M	M				
Donor BMI (kg/m^2)	25.8	33.8	23.5	29.2				
Donor HbA_{1c} or other measure of blood glucose control	5.0	5.4	5.2	5.4				
Origin/source of islets[b]	Donate Life California Organ and Tissue Donor Registry	Donate Life California Organ and Tissue Donor Registry	Donate Life California Organ and Tissue Donor Registry	Donate Life California Organ and Tissue Donor Registry				
Islet isolation centre	Prodo Labs	Prodo Labs	Prodo Labs	Prodo Labs				
Donor history of diabetes? Please select yes/no from drop down list	No	No	No	No				
If Yes, complete the next two lines if this information is available								
Diabetes duration (years)								
Glucose-lowering therapy at time of death[c]								
RECOMMENDED INFORMATION								
Donor cause of death	Stroke	Head trauma	Head trauma	Stroke				
Warm ischaemia time (h)	0.25	0.5	0.5	0				
Cold ischaemia time (h)	11	8	9.25	7.5				
Estimated purity (%)	95	95	95	80				
Estimated viability (%)	95	95	95	85				
Total culture time (h)[d]	120	108	108	120				
Glucose-stimulated insulin secretion or other functional measurement[e]	N/A	N/A	N/A	N/A				

Handpicked to purity? Please select yes/no from drop down list	Yes	Yes	Yes	Yes				
Additional notes								
